# Sensory modulation and post meal distress at Royal Melbourne Hospital Eating Disorders Specialist Unit

**DOI:** 10.1186/2050-2974-2-S1-P4

**Published:** 2014-11-24

**Authors:** Rebecca Clarke

**Affiliations:** 1 Royal Melbourne Hospital Eating Disorders Specialist Unit, Melbourne, Australia

## 

Sensory modulation utilises sensory input from external senses (vision, taste, smell, hearing and touch) and internal senses (vestibular, proprioceptive and deep pressure touch), along with abdominal breathing. It has been shown to help with stress reduction, relaxation, emotional regulation and symptom management of adolescent consumers and elderly consumers. An exploratory method was used to evaluate if Sensory Modulation had an impact on reducing post meal distress in adult consumers suffering from eating disorders. The data was collected from inpatient eating disorder consumers. There ranged between five and eight consumers per group per week. The Sensory Modulation Group ran once a week for forty five minutes over a ten week period. The Sensory Modulation Consumer Self Rating Tool was used to rate the consumer mood before and after Sensory Modulation was implemented. It was found that Sensory Modulation utilised after a meal reduced post meal distress by 10%-20%. With increased implementation on the inpatient unit, Sensory Modulation allows inpatient consumers to find a sensory modality that works for them that they can utilise in the community to manage post meal distress.

